# A Practical and Validated Fall Risk Screening Instrument: A Systematic Review

**DOI:** 10.1093/geroni/igaa057.752

**Published:** 2020-12-16

**Authors:** Wytske Meekes, J C Korevaar, C J Leemrijse, L A M van de Goor

**Affiliations:** 1 Tilburg University, Tilburg, Noord-Brabant, Netherlands; 2 Nivel (Netherlands Institute for Health Services Research), Utrecht, Utrecht, Netherlands

## Abstract

Early detection of a high fall risk is important to start fall preventive interventions in time and to reduce fall risk among older people. Several fall risk screening instruments are available, however it is unclear which instrument is validated and most suitable for the primary care setting. This systematic review aims to identify the most suitable fall risk screening instrument(s) for the primary care setting (i.e. requires limited time, no expensive equipment and no additional space) with good prognostic ability to assess high fall risk among independently living older people. An extensive search was conducted in the databases PubMed, EMBASE CINAHL, Cochrane and PsycINFO. Twenty-six out of 2277 articles published between January 2000 and February 2019 were included. Six fall risk screening instruments were identified; TUG test, Gait Speed test, BBS, POMA, FR test, Fall History. Most articles reported AUCs ranging from 0.5-0.7 for all instruments. Sensitivity and specificity varied substantially across studies (e.g. TUG, sens.: 10-83.3%, spec.:37-96.6%). The results showed that none of the included screening instruments had sufficient (AUC>0.7) predictive performance (Šimundić, 2009). As suitability for the primary care setting prevails for now, Fall History appears to be the most suitable screening instrument. Compared to the other instruments, Fall History requires the least amount of time, no expensive equipment, no training, and no space (adjustments). Patient’s fall history together with a health care professional’s clinical judgment, might be a promising screening strategy for the primary care setting to identify high fall risk among older people.

